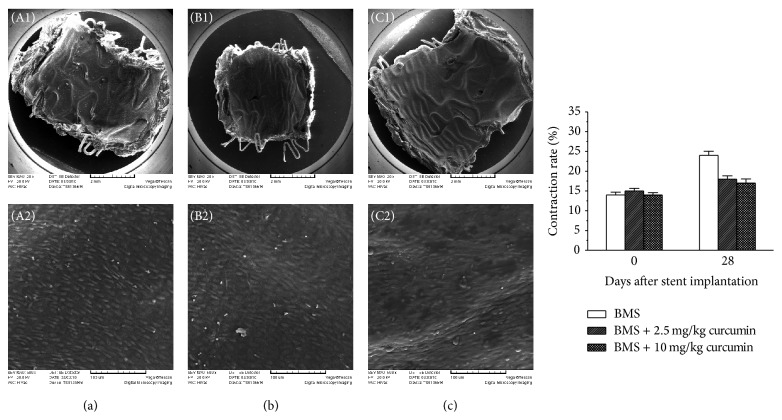# Accelerated Recovery of Endothelium Function after Stent Implantation with the Use of a Novel Systemic Nanoparticle Curcumin

**DOI:** 10.1155/2015/291871

**Published:** 2015-06-08

**Authors:** Qi Lu, Fang Ye, Xiangjun Yang, Qingqing Gu, Peng Wang, Jianhua Zhu, Li Shen, Feirong Gong

**Affiliations:** ^1^Department of Cardiology, Affiliated Hospital of Nantong University, Nantong 226001, China; ^2^Department of Radiology, Zhongshan Hospital, Fudan University, Shanghai 200032, China; ^3^Department of Cardiology, First Affiliated People's Hospital of Suzhou University, Suzhou 215006, China; ^4^Department of Electrocardiogram, First Affiliated People's Hospital of Suzhou University, Suzhou 215006, China; ^5^Key Laboratory for Ultrafine Materials of Ministry of Education, School of Materials Science and Engineering, East China University of Science and Technology, Shanghai 200237, China

## Abstract

Curcumin was reported to exhibit a wide range of pharmacological effects including antioxidant, anti-inflammatory, and antiproliferative activities and significantly prevent smooth muscle cells migration. In the present study, a novel kind of curcumin loaded nanoparticles (Cur-NP) has been prepared and characterized with the aim of inhibiting inflammation formation and accelerating the healing process of the stented arteries. Cur-NP was administrated intravenously after stent implantation twice a week and detailed tissue responses were evaluated. The results demonstrated that intravenous administration of Cur-NP after stent implantation accelerated endothelial cells restoration and endothelium function recovery and may potentially be an effective therapeutic alternative to reduce adverse events for currently available drug eluting stents.

## 1. Introduction

Drug eluting stents (DES) have been attracting tremendous attention through remarkable reduction of angiographic target lesion revascularization [1]. In the past few years, in-stent restenosis rates following DES placement have been reported to be typically less than 10% [2, 3]. In real world clinical settings, however, concerns have arisen regarding adverse effects of DES in some patients, including incomplete neointimal coverage, impaired endothelial cell function, thrombosis, hypersensitivity reactions, and incomplete stent apposition [4–7]. A lack of full endothelial strut coverage in any given section was reported to be the best single correlate of thrombosis [8]. Based on the correlation between reendothelialization and long-term implantation results, it has been supposed that a rapid restoration of functional endothelium may provide a possible approach to improving long-term safety and efficacy of drug eluting stents [9].

Curcumin, a polyphenolic natural extract of* Curcuma longa*, exhibits a wide range of pharmacological effects including antioxidant, anti-inflammatory, and antiproliferative activities in various preclinical models [10–13]. Curcumin was also reported to effectively prevent smooth muscle cells migration and significantly reduce polymer-induced cell inflammatory responses [14, 15]. Moreover, curcumin has been a daily food of the South Asians for thousands of years which defends its innocuity, and to date, studies in both animals and humans have confirmed that curcumin is well avirulent, bioactive, and innocuous even with a daily intake of 10 g [16, 17]. Nevertheless, widespread clinical applications of this quite efficacious agent in the treatment of diseases have been limited due to its water insolubility (~13.76 *μ*g/mL) and instability at physiological conditions and consequently rapid metabolism and excretion and minimal systemic bioavailability. Thus the prerequisite in the design of an efficient curcumin carrier is to stably retain the loaded drug in the blood circulation before accessing the target site.

Among the drug delivery systems, polymeric nanoparticles have been investigated extensively for their drug loading capacity and ability to deliver hydrophobic drugs* in vivo*. One the other hand, polymeric nanoparticle was more stable than other nanodrug delivery systems such as liposome and polymeric micelles, which was suitable for* in vivo* curcumin delivery. In the present study, a novel kind of curcumin loaded biodegradable nanoparticles has been prepared and characterized. With the assistance of block copolymer, curcumin can be encapsulated into water dispersible nanoparticles and be intravenously injectable. The purpose of the present study was to obtain detailed systematic insights into the tissue responses to curcumin administration following stent implantation. We hypothesized that administration of curcumin nanoparticles (Cur-NP) reduces in-stent restenosis, inhibits inflammation formation, and accelerates the healing process after stent implantation and may potentially be an effective therapeutic alternative to reduce safety problems for drug eluting stents.

## 2. Materials and Methods

### 2.1. Materials

Methoxy-poly(ethylene glycol) with molecular weights of 2000 and 5000, 3-(4,5-dimethyl-2-thiazolyl)-2-5-diphenyl tetrazolium bromide (MTT), and stannous octoate (Sn(Oct)_2_) were obtained from Sigma-Aldrich (St. Louis, MO, USA). DL-Lactide (DL-LA, PURAC Biochem, Netherlands) was recrystallized twice from anhydrous ethyl acetate and dried under vacuum at room temperature. Curcumin (Cur) was synthesized from our laboratory with a purity more than 99.5%. Bare metal stents (BMS, 3.0 × 17 mm) were kindly given by Beijing Amsinomed Medical Company, China. All other chemicals were of analytical grade and used without further purification.

### 2.2. Animals

Three-month-old male pigs (~15 kg weight) were obtained from Shanghai Animal Administration Center and received daily oral antiplatelet medication until termination. All animal experiments were approved by the Animal Care and Use Committee of Fudan University and were in compliance with the “Guide for the Care and Use of Laboratory Animals” published by the National Academy Press (NIH Publication number 85-23, revised in 1996).

### 2.3. Synthesis of Methoxy-poly(ethylene glycol)-poly(D,L-lactide) (mPEG-PDLLA) Block Copolymer

A ring opening polymerization of DL-lactide using Sn(Oct)_2_ as a catalyst and mPEG as a macroinitiator was employed to synthesize the mPEG-PDLLA diblock copolymer [18–21]. Briefly, mPEG_5000_ (1 g) was added in a Schlenk bottle and degassed at 130°C under reduced pressure with magnetic stirring for 3 h to eliminate the water content. Then DL-lactide (4 g) and Sn(Oct)_2_ (4 mg) were added and the bottle was flame-sealed under a vacuum. The mixture was then stirred at 130°C for 24 h. The reaction product was recovered by dissolving in dichloromethane (DCM), followed by precipitation in cold ether. The resultant white powder precipitate was filtered and obtained by vacuum drying at room temperature for three days and then stored at 4°C for further use. ^1^H-NMR analysis was performed on a Bruker ARX-400 spectrometer to determine the number average molecular weight of the two blocks. The final molecular weight of the mPEG block was 5000 g/mol and the molecular weight of the PDLLA block was 17000 g/mol with a polydispersity of 1.62.

The mPEG_2000_-PDLLA_1800_ block copolymer was synthesized with the same procedure and used as the surfactant in the preparation of nanoparticles. The feed molar ratio of mPEG_2000_ and DL-lactide was fixed to 1 : 1.2 and the final molecular weight of the mPEG block was 2000 g/mol and the molecular weight of the PDLLA block was 1800 g/mol with a polydispersity of 1.05.

### 2.4. Preparation and Characterization of Curcumin Loaded Nanoparticles (Cur-NP)

Curcumin loaded nanoparticles (Cur-NP) were prepared by a single-emulsion method (otherwise called nanoemulsification) [22] with some modification. Briefly, mPEG_5000_-PLA_17000_ (150 mg) and curcumin (30 mg) were dissolved in dichloromethane (15 mL) and combined with a water phase (150 mL) containing 0.2% mPEG_2000_-PLA_1800_ as the surfactant under stirring at 2800 rpm. After that, the stirring speed was increased to 3700 rpm within 2 minutes and maintained for about 5 min. The Cur-NP containing solution was then gently stirred at room temperature overnight to evaporate the solvent. The resulting Cur-NP suspension was purified and concentrated with ultrafiltration (Millipore; molecular weight cutoff is 300 kDa). Finally, the nanoparticle solution was lyophilized and stored at 4°C for further use. The morphologies of the Cur-NP were observed under transmission electron microscope (TEM, JEM-2100, JEOL, Japan). The mean diameter and particle size distribution of the nanoparticles were determined with a PSS Nicomp 380ZLS particle sizer (Particle Sizing Systems, USA) by dynamic light scattering method. The encapsulation efficiency and drug loading capacity of the nanoparticles were determined by the UV-Visible Spectrophotometer (UV-2450, Shimadzu, Japan) at 425 nm using the calibration curve constructed earlier. The drug encapsulation efficiency (EE) and drug loading capacity (DLC) were calculated based on the following formulas:

(1) Encapsulation efficiency (EE)=weight of drug in micelles weight of the initial drug ×100%,Drug loading capacity (DLC)=weight of drug in micelles weight of micelles×100%.

The thermal properties of free curcumin, mPEG_5000_-PDLLA_17000_, and Cur-NP were studied using a differential scanning calorimeter (DSC) (DMA Q800, USA). Samples were heated from 20°C to 250°C under nitrogen atmosphere at a heating rate of 10°C/min.

### 2.5. *In Vitro* Stability of the Reconstituted Nanoparticles

The* in vitro* stability of the reconstituted Cur-NP was evaluated in aqueous solution at 37°C. Due to the low aqueous solubility, the released curcumin crystalized and precipitated subsequently in the solution. In detail, the initial concentration of curcumin was adjusted to 2 mg/mL by ultrapure water. At predetermined time intervals, the mean particle size and polydispersity (PI) were monitored by DLS. In addition, samples were taken from the solution and centrifuged at 8,000 rpm for 10 min to remove the precipitate, and then the concentrations of curcumin still solubilized in the solution were determined.

### 2.6. *In Vitro* Curcumin Release Profile

A modified dialysis method was used to investigate the* in vitro* release behavior of Cur from Cur-NP. In detail, free Cur in ethanol or Cur-NP in water (initial curcumin concentration = 2 mg/mL) were placed in dialysis tubes (molecular weight cutoff is 8–14 kDa). The dialysis tubes were incubated in 200 mL of prewarmed water containing ethanol (40 wt%) with gentle shaking (100 rpm) at 37°C. At specific times, 4 mL of the release media was removed and replaced by prewarmed fresh release media. Curcumin concentration in the media was quantified using UV-Visible Spectrophotometer and the release profile was plotted. All the results were the mean of three test runs and all data were expressed as the mean ± SD.

### 2.7. Cell Studies

Freshly isolated rat vascular smooth muscle cells (rVSMC) from rat aortas were kindly given by Professor Shen and cultured in Dulbecco's modified Eagle's medium (DMEM, Gibco) supplemented with 20% fetal bovine serum (FBS, Gibco) in a humidified incubator equilibrated with 5% CO_2_-95% air for 4–6 days. The cytotoxic effect of curcumin on rVSMC was investigated using 3-(4,5-dimethylthiazol-2-yl)-2,5-diphenyl tetrazolium bromide (MTT) assay [23]. Briefly, cells were seeded in 96-well plates at a density of 5,000 cells per well and contacted with various concentrations of curcumin nanoparticles (final curcumin concentration = 2, 5, 10, 20, 40, and 60 *μ*g/mL) for 24 h, 48 h, and 72 h, respectively. 50 *μ*L of MTT (5 mg/mL in 0.01 M PBS, pH 7.4) was then added to each well and incubation continued at 37°C for an additional 4 h in the dark. The medium was then carefully replaced with 150 *μ*L DMSO, followed by agitating thoroughly for 10 min in a thermoshaker (MB100-2A, China), and read on a microplate reader (Molecular Devices, USA) at a wavelength of 490 nm. The reduction in optical density was used as a measurement of cell viability, normalized to cells incubated in control medium, which were considered 100% viable.

### 2.8. Stent Implantation

On the procedure day, nine three-month-old male pigs weighing about 15 kg each were divided into three groups and anesthetized with ketamine (20 mg/kg intramuscularly) and xylazine (2 mg/kg intramuscularly). One group (*n* = 3) received BMS implantation singly. The other two groups (*n* = 3 for each group) received the combination therapy of BMS implantation and curcumin nanoparticles administration intravenously every 3 days and twice a week (q3d × 2) at the dosage of 2.5 mg/kg and 10 mg/kg, respectively. The pigs receiving two stents each in the left anterior descending artery (LAD) and right coronary artery (RCA) and 18 stents were used in the animal experiment. The resulting stent-to-artery ratio was about 1.2–1.3 : 1 by quantitative coronary angiography analysis. The animals were anesthetized with ketamine (20 mg/kg) and xylazine (2 mg/kg) for follow-up angiography in the same orthogonal views before death with 20 mL of potassium chloride intracoronary injection. Then the stented arteries were carefully dissected from the myocardium and cut into two pieces equally, each about 9 mm long for cross sections preparation and SEM imaging.

Endothelium function after stent implantation was estimated by measuring the coronary vasomotor reactivity in response to acetylcholine (Ach, 60 mg, performed at an infusion rate of 1 mL/min) infusion after 28 days [24].

### 2.9. Histological Analysis of Neointimal Hyperplasia

The areas enclosed by internal elastic lamina (IEL, mm^2^) and the lumen area (LA, mm^2^) were measured. Neointimal area (NA, mm^2^) was calculated as follows: NA = IEL − LA. The percent neointimal stenosis was calculated using the following equation: % stenosis = NA/IEL × 100%.

### 2.10. Statistical Analysis

Numerical data are presented as mean ± standard error of the mean. Continuous variables were compared by ANOVA (*t*-test with Bonferroni correction), and categorical variables were compared by *χ* ^2^ test. A *P* value of ≤0.05 was considered as a significant difference.

## 3. Results and Discussion

### 3.1. Preparation and Characterization of Cur-NP

**Figure 1:**
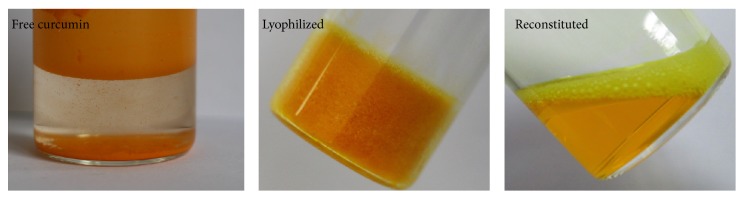
The appearances of free curcumin in aqueous, lyophilized and reconstituted curcumin nanoparticles. Curcumin concentration: 2 mg/mL.

**Figure 2:**
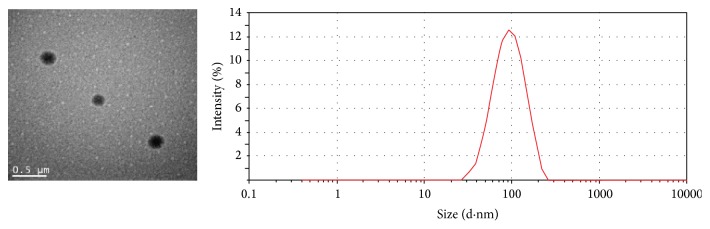
TEM image (left) of curcumin nanoparticles and particle size distribution (right) measured by dynamic light scattering method.

Curcumin was easily encapsulated by a nanoemulsification method in the presence of mPEG_2000_-PLA_1800_ as the surfactant, which is simple and easy for scale-up preparation. In this process, the hydrophobic curcumin was physically trapped within the core of the nanoparticles by hydrophobic interaction with PDLLA block. The EE and DLC of the prepared Cur-NP were 95 ± 2.5% and 17 ± 0.68%, respectively. The prepared Cur-NP was easy to reconstitute in aqueous media including pure water and saline by simply shaking to form a clear solution, and the resultant solution was very stable without any precipitation formed even after more than one week of storage at 37°C. The appearances of the lyophilized Cur-NP and reconstituted nanoparticles in water were presented in Figure 1. It is clear that the lyophilized Cur-NP were completely dispersed in water. The TEM photograph (Figure 2) showed that Cur-NP have regular spherical shape with a monodisperse distribution. As shown in Figure 2, the mean diameter and the polydispersity of the reconstituted Cur-NP were 98 nm and 0.24, respectively. Also no significant difference in diameter and polydispersity of the nanoparticles after lyophilization was observed, which suggested that Cur-NP were quite stable during the lyophilization process.

**Figure 3:**
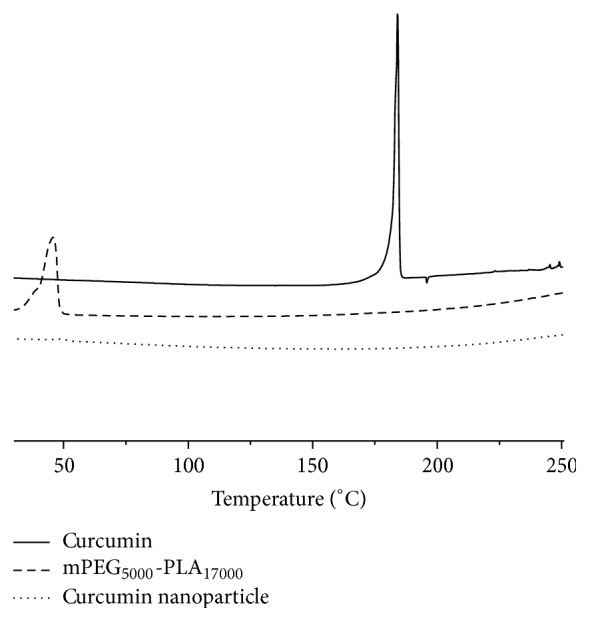
Differential scanning calorimetry (DSC) thermograms of free curcumin, mPEG_5000_-PLA_17000_, and curcumin nanoparticles.

The differential scanning calorimetry (DSC) thermograms of free curcumin and lyophilized Cur-NP were shown in Figure 3. The melting point of curcumin crystal was about 182°C, while in the DSC curve of Cur-NP, the intrinsic melting transition peak of curcumin vanished, indicating that all curcumin has been molecularly incorporated into the core of the nanoparticles, which also explained well that nanoparticle encapsulation could greatly enhance the solubility of curcumin.

### 3.2. *In Vitro* Stability and* In Vitro* Curcumin Release Profile of the Reconstituted Cur-NP

**Figure 4:**
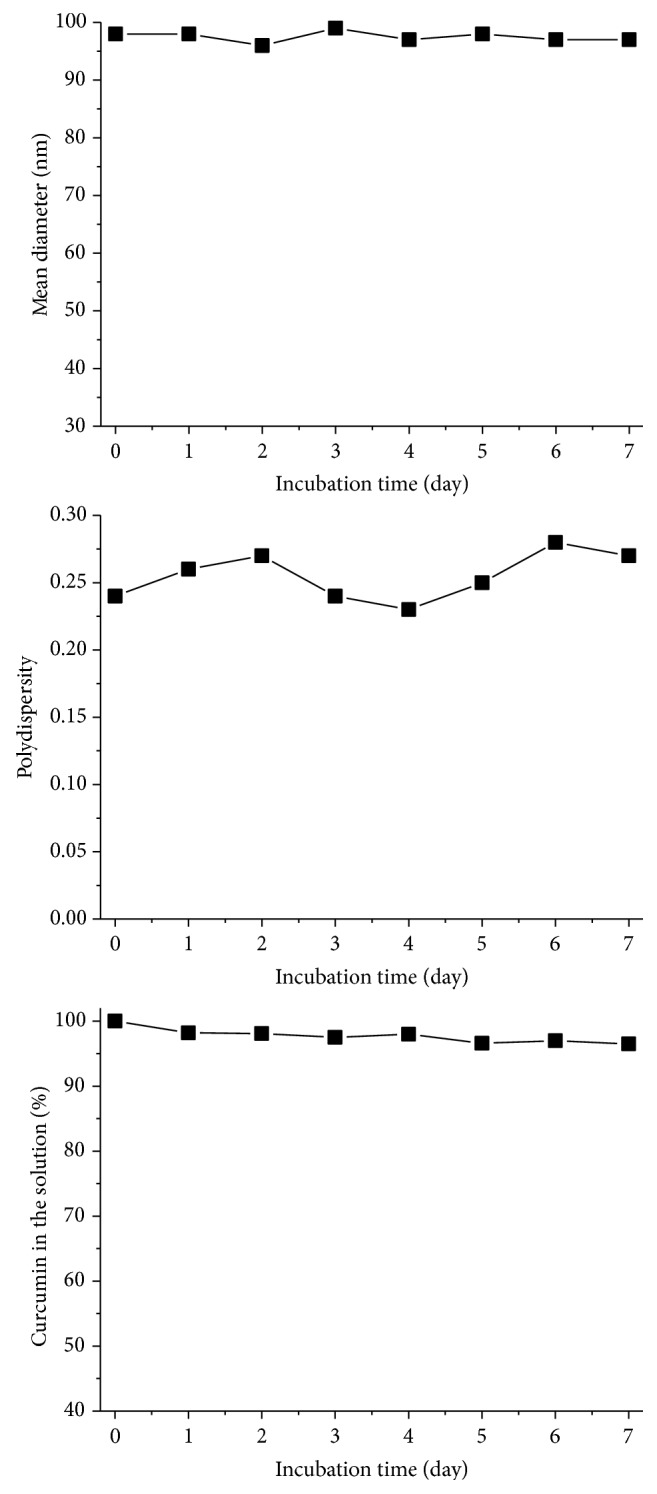
Mean particle size, polydispersity, and curcumin in the solution of reconstituted curcumin nanoparticles as a function of standing period in aqueous solution at 37°C; initial curcumin concentration: 2 mg/mL.

There is a close relationship between the stability of the drug loaded nanoparticles and their* in vivo *performance, such as biodistribution and targeting. To evaluate the stability of Cur-NP prepared in this study, we incubated this kind of nanoparticle curcumin in aqueous solution at 37°C for one week and analyzed their size, PI, and drug concentration changes over time by DLS and UV-Visible absorption spectra. As demonstrated in Figure 4, during the incubation, almost no changes happened in the size and PI for the nanoparticles and the concentration of curcumin in the solution remained constant.

**Figure 5:**
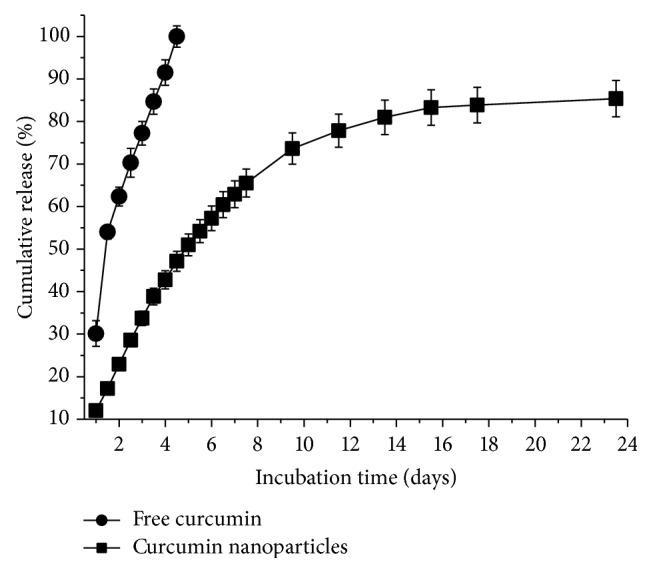
The release profiles of free curcumin and curcumin nanoparticles at 37°C. The release medium was prewarmed ultrapure water containing 40% (v/v) of ethanol. Each piece of data represents the mean ± standard deviation (*n* = 3).

The* in vitro* release profile of free curcumin and Cur-NP was investigated by a modified dialysis method with 40 wt% ethanol solution as the release medium. As shown in Figure 5, only about 40% of curcumin was released from Cur-NP within the first 4 h, while almost 100% of curcumin was released from free curcumin during the same time period. After 24 h, about 20% of the initially incorporated curcumin still existed in the nanoparticles. The results indicated that the nanoparticle formulation showed a sustained-release property for the incorporated curcumin.

### 3.3. Cytotoxicity of Curcumin on rVSMC

**Figure 6:**
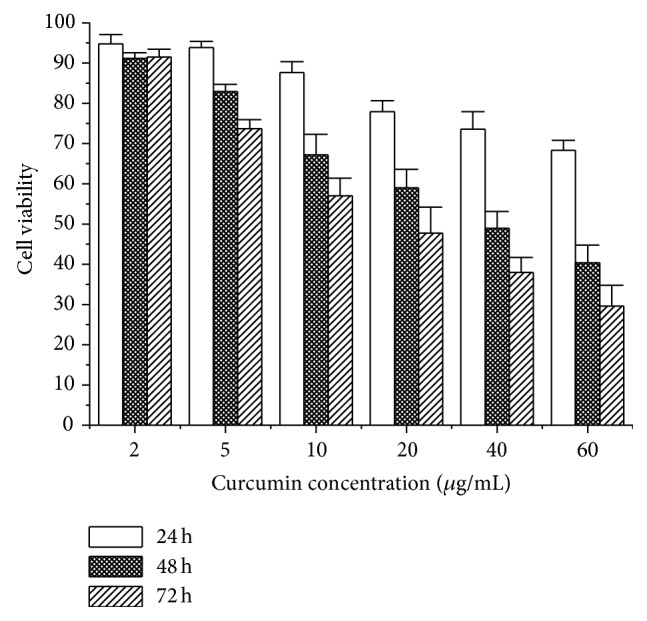
The cell viability after 24, 48, and 72 h of incubation with various concentrations of curcumin nanoparticles. Initial curcumin concentrations were 2, 5, 10, 20, 40, and 60 *μ*g/mL.

VSMC proliferation is a major reason for neointimal hyperplasia after stent implantation [25, 26]. It is therefore critical to inhibit the proliferation of VSMC in order to reduce in-stent restenosis after stent implantation. To investigate whether Cur-NP could be used to effectively deliver the hydrophobic curcumin to cells, a comparative cytotoxicity study of Cur-NP to rat smooth muscle cells was performed with MTT assay. As demonstrated in Figure 6, curcumin had a dose-dependent cytotoxic effect on rVSMC. After 24, 48, and 72 h incubation with 2, 5, 10, 20, 40, and 60 *μ*g/mL curcumin, cell viability was 94.8 ± 2.3%, 93.9 ± 1.5, 87.6 ± 2.7, 77.9 ± 2.8, 73.6 ± 4.3, and 68.3 ± 2.5; 91.2 ± 1.4, 82.9 ± 1.8, 67.2 ± 5.1, 59 ± 4.6, 48.9 ± 4.2, and 40.4 ± 4.4; and 91.5 ± 1.9, 73.7 ± 2.2, 57 ± 4.4, 47.7 ± 6.5, 38 ± 3.7, and 29.6 ± 5.2, respectively, of the control level. The other way round, mPEG_5000_-PLA_17000_ nanoparticles enhanced the solubility of curcumin effectively and provided a convenient way for cellular uptake.

### 3.4. Morphological Evaluation of the Stented Arteries

**Figure 7:**
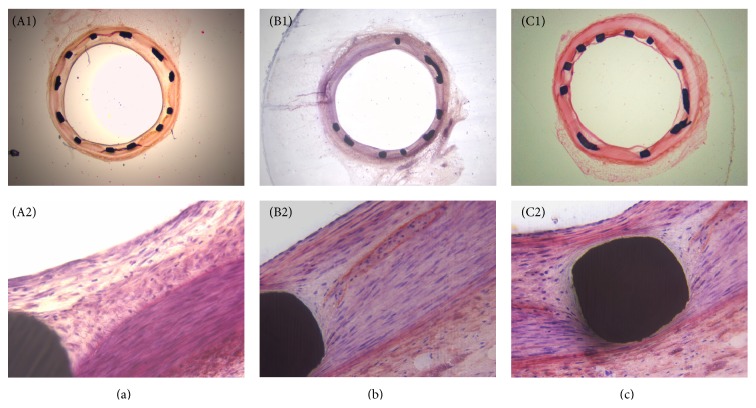


**Figure 8:**
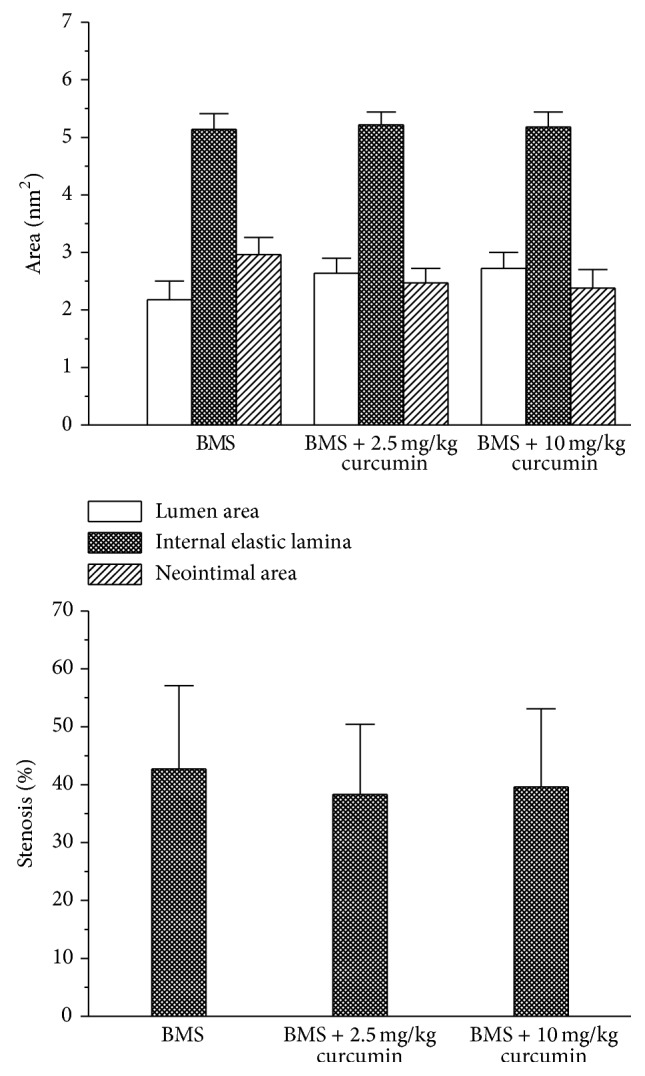
Histomorphometric analysis of the stented arteries. At 28 days after deployment, arteries with stents were prepared, stained, and analyzed.

In the animal experiment, a complete blood cell count showed similar results for white blood cell count, platelet count, and hematocrit among all groups and no toxicity was observed throughout the experiment. At 28 days after stent deployment, cross section slices of the stented arteries were prepared and stained with hematoxylin and eosin for measuring vessel area and performing histological analysis. The typical morphologies of the stented arteries are shown in Figure 7. No distinctive vessel narrowing and remodeling effect on the surrounding tissues could be observed. Also no obvious inflammation responses were found in each group. As summarized in Figure 8, the percent area stenosis for BMS, BMS + 2.5 mg/kg curcumin, and BMS + 10 mg/kg curcumin at 28 days was 42.7 ± 14.4%, 38.3 ± 12.1%, and 39.6 ± 13.5%, respectively, and the difference was not significant (*P* > 0.05), although the combination therapy of BMS and Cur-NP had slightly lower neointimal area.

### 3.5. Endothelialization of the Stented Arteries and Evaluation of Endothelium Function

Endothelial recovery is an essential component for vascular healing by providing critical structural and antithrombogenic functions [27]. The endothelialization of the stented arteries was examined using SEM at 28 days after stent implantation as shown in Figure 9. Both the amount and the morphology of the endothelial cells on the stent surfaces were analyzed. At this time point, all the arteries treated with BMS with or without Cur-NP administration were fully endothelialized and the lumen surface of the vessel wall and the stent struts were covered by confluent endothelial cells. The cells on the BMS surfaces without Cur-NP administration appeared to be infantile and undeveloped, while in the combination therapy, the luminal surface and the stent struts had been covered with confluent shuttle-like endothelial cells and the cells seemed to be more integrated. They formed a continuous mat which was aligned with the direction of the flow of blood.

Coronary stenting leads to disruption of the endothelial layer and leaves a thrombogenic metallic surface exposed to the blood stream [28], facilitating the Cur-NP accumulation in the stented arteries similarly to the enhanced permeability and retention (EPR) effect in cancer therapy [29]. Then curcumin released from the nanoparticles with a sustained manner in the stented arteries decreases inflammation formation and may accelerate the healing process. Endothelium function evaluation was prospectively designed to compare coronary endothelial dysfunction between the stented arteries as well as morphology observation. The combination therapy seemed to accelerate the endothelial cell restoration from the SEM images; also, in this study, there was a trend that the segments distal to the stents in the single therapy should be more strongly constricted to the Ach infusion at 28 days (Figure 9), and the difference was significant (*P* < 0.05). The results indicated that rapid restoration of endothelial cells on the stent surface might mean rapid endothelium function recovery, which may be associated with long-term clinical benefits, although the truly healing process after stent implantation may take much longer time than the period of endothelial cell restoration on the stent surfaces.

## 4. Conclusions

In conclusion, these studies demonstrated that the combination therapy of Cur-NP administration following stent implantation can enhance endothelial cells restoration and endothelium function recovery and may potentially be an effective therapeutic alternative to improve safety of currently available drug eluting stents.

## Figures and Tables

**Figure 9:**